# Fabrication of Nano Iron Oxide–Modified Biochar from Co-Hydrothermal Carbonization of Microalgae and Fe(II) Salt for Efficient Removal of Rhodamine B

**DOI:** 10.3390/nano12132271

**Published:** 2022-07-01

**Authors:** Ziling Peng, Zeyu Fan, Xia Chen, Xian Zhou, Zhuo Fan Gao, Shanshan Deng, Sha Wan, Xingdong Lv, Yan Shi, Wei Han

**Affiliations:** Changjiang River Scientific Research Institute, Research Center of Water Engineering Safety and Disaster Prevention of Ministry of Water Resources, Wuhan 430010, China; pengziling0304@163.com (Z.P.); zhouxian@mail.crsri.cn (X.Z.); gaofz@mail.crsri.cn (Z.F.G.); dengss@mail.crsri.cn (S.D.); wansha@whu.edu.cn (S.W.); lvxingdong9010@126.com (X.L.); 18163559730@163.com (Y.S.); hanwei@mail.crsri.cn (W.H.)

**Keywords:** nano iron oxide, biochar, magnetic, Rhodamine B, adsorption

## Abstract

Dye adsorption by magnetic modified biochar has now received growing interest due to its excellent adsorption performance and facile separation for recycling. In this study, nano iron oxide–modified biochar was fabricated via the successive hydrothermal-pyrolyzing method using *Chlorella vulgaris* (Cv) and FeSO_4_·7H_2_O as raw materials, and its adsorption on Rhodamine B (RhB) in aqueous solution was studied. Multiple techniques such as X-ray diffraction (XRD), Fourier transform infrared spectroscopy (FTIR), scanning electron microscopy (SEM), transmission electron microscopy (TEM), Brunauer–Emmett–Teller (BET), vibrating sample magnetometry (VSM) and X-ray photoelectron spectroscopy (XPS) were employed to comprehensively characterize the structure, morphology and physicochemical properties of the adsorbent. The as-synthesized nano iron oxide–modified biochar (CBC-Fe(II)) exhibited a large surface area (527.6 m^2^/g) and high magnetic saturation value (13.7 emu/g) to facilitate magnetic separation. Compared with CBC and CBC-Fe(III), CBC-Fe(II) exhibited superior adsorption ability towards RhB in aqueous solution, with a maximum adsorption capacity of 286.4 mg/g. The adsorption process of RhB onto CBC-Fe(II) was well described by the pseudo-second-order kinetic model and Langmuir isotherm model, indicating monolayer chemisorption behaviors for the adsorption system. Facile preparation, great adsorption performance and magnetic recovery properties endow CBC-Fe(II) to be a promising adsorbent for dye removal.

## 1. Introduction

Recently, the content of organic pollutants in the environment has been increasing due to the rapid development of industry, leading to severe environmental issues [1,2,3,4]. Specifically, the disposed water from the textile, printing, cosmetics, medicine and other industries contains a significantly high amount of organic dye molecules which are potentially carcinogenic and teratogenic with high toxicity, posing a serious threat to various organisms and human health [5,6]. Therefore, developing an efficient technology to remove those dye contaminates from wastewater is indispensable to prevent environmental pollution.

A variety of treatment techniques have been utilized for the removal of dyes from polluted water, including coagulation [7], flocculation [8], oxidation [9], adsorption [10], electrochemical separation [11], microbiological decomposition [12] and the membrane process [13]. Among all, adsorption has become one of the most widely used methods due to its advantages of low cost, high efficiency, simple operation and environment-friendliness [14,15,16]. Some adsorbents developed for removing dyes from wastewater include bentonite [17], graphene [18], activated carbon [19], etc., and research is still going on to develop more economical, sustainable and efficient materials as new adsorbents for dye removal.

Biochar (BC), a carbon-based porous product obtained from the pyrolysis of biomass and possesses high potential for dye pollution remediation owing to its renewability, stability, its various sources, environmental sustainability and its beneficial physicochemical properties, including high surface area, developed pore structure, and others [20,21]. The renewable resources can be easily obtained from agricultural waste or naturally abundant biomass such as fruit peels, coconut shells, cassava slag, rice husk, sewage sludge, macroalgae, microalgae, etc. [22,23,24]. Wu et al. [25] prepared biochar from agricultural waste cassava slag by hydrothermal carbonization treatment, and the cassava slag biochar had excellent adsorption performance on RhB with a maximum adsorption capacity of 105.3 mg/g. Hou et al. [26] synthesized biochar with well-developed pores using agricultural waste from bamboo shoot shells as the raw materials. The biochar produced at the carbonization temperature of 800 °C could efficiently remove RhB from water. Vigneshwaran et al. [27] fabricated sulfur-doped biochar derived from tapioca peel waste, which had the adsorption capacity of 30.18 and 33.10 mg/g for malachite green and RhB dye, respectively. Nevertheless, the adsorption capacity of the above reported biochar is relatively limited. Moreover, solid–liquid separation often requires complementary steps of centrifugation and filtration, restraining its application potential.

In this case, biochar activated by magnetic modification exhibits controlled movement under an external magnetic field, making it easy to separate [28]. Magnetic biochars are fabricated by chemically combining them with other functional elements such as Fe, Fe_3_O_4_, γ-Fe_2_O_3_, CoFe_2_O_4_, etc., which leads to strong metal binding [29,30,31]. Though magnetic biochar has the advantage of easy separation, the surface pores are prone to be plugged with those functional particles, causing a decrease in its adsorption efficiency [32]. In addition, Fe-modified biochar is widely applied in the advanced oxidation process and the adsorption of heavy metal ions, but there are few studies involving adsorption of dyes by Fe-modified biochar [33]. Therefore, it is essential to explore a facile method to fabricate magnetic biochar adsorbents for dye removal and improve adsorption properties of biochar in the meantime.

Herein, nano iron oxide–modified biochar was successfully prepared via successive hydrothermal and pyrolyzing treatments. One of the easily cultivated microalgae (Cv) was selected as carbonaceous feedstock in this work owing to the wide abundance, high biomass productivity as well as the technical challenges associated with its treatment and disposal [34,35,36]. The obtained nano iron oxide–modified biochar was analyzed via XRD, FTIR, SEM, TEM, BET, VSM and XPS techniques. RhB, as a representative cationic organic dye compound, is considered to be more difficult to remove due to the high solubility in water [37,38]. Thus, RhB dye was chosen as the target contaminant to assess the adsorption efficiency of the nano iron oxide–modified biochar. The influence of adsorbent dosage, initial RhB concentration, adsorption time and temperature on the adsorption performance was also examined. The mechanism of RhB adsorption was further analyzed via kinetic and isothermal models. The results of this study offer a new approach to dye wastewater treatment using nano iron oxide–modified biochar and explore a new way for the resource utilization of Cv.

## 2. Materials and Methods

### 2.1. Materials

Cv powder was obtained from Xi’an Youshuo Biotechnology Co., Ltd. (Xi’an, China) Iron(II) sulfate heptahydrate (FeSO_4_·7H_2_O), iron(III) nitrate nonahydrate (Fe(NO_3_)_3_·9H_2_O), nitric acid (HNO_3_), ethanol (C_2_H_5_OH) and RhB were all supplied by Sinopharm Chemical Reagent Co., Ltd. (Shanghai, China) All chemical reagents were of analytical grade and were used without further purification. Ultrapure water (18.25 MΩ·cm) was used in all the experiments.

### 2.2. Preparation of Adsorbents

#### 2.2.1. Preparation of Biochar

The *Chlorella*-derived biochar (CBC) was prepared through pyrolyzing the carbonaceous precursor (Cv). First, the Cv powder was dried at 60 °C overnight. Then the dried precursor was heated at 5 °C/min to 900 °C in a tubular furnace under nitrogen atmosphere and carbonized for 1 h. After cooling to room temperature, the obtained product was ground in a ball mill for 8 h. Finally, the powder was washed separately with HNO_3_, absolute ethyl alcohol and ultrapure water and then dried at 60 °C to obtain pure CBC.

#### 2.2.2. Preparation of Nano Iron Oxide–Modified Biochar

Nano iron oxide–modified biochar was prepared via successive hydrothermal-pyrolyzing method. Two kinds of iron salts with different valences (FeSO_4_·7H_2_O and Fe(NO_3_)_3_·9H_2_O) were selected as iron sources. First, a certain amount of Cv powder was dispersed in a FeSO_4_·7H_2_O solution under continuous stirring in nitrogen atmosphere. The mass ratio of Fe element to Cv was fixed at 1:6. The mixture was then placed into a Teflon-lined autoclave and hydrothermally treated at 150 °C for 24 h. After the resulting product was dried at 60 °C, it was pyrolyzed at 900 °C using a similar procedure as preparing CBC. Then the obtained product was ball milled for 8 h. Additionally, to remove metallic and organic impurities, the powder was washed separately with HNO_3_, absolute ethyl alcohol and ultrapure water. At last, the sample was dried at 60 °C to yield nano iron oxide–modified biochar, which was denoted as CBC-Fe(II). For comparison, CBC-Fe(III) was also prepared with the similar procedure using the iron salt of Fe(NO_3_)_3_·9H_2_O instead of FeSO_4_·7H_2_O.

### 2.3. Characterizations

The obtained samples were characterized by XRD analysis on D8 advance X-ray diffractometer (Bruker AXS, Karlsruhe, Germany) with filtered Cu Kα radiation. The functional groups of the samples were analyzed by FTIR (Thermo Scientific, Nicolet 67000, Madison, WI, USA). The detailed microstructure and morphology of samples were observed using SEM (ZEISS Gemini SEM 300, Oberkochen, Germany) and TEM (FEI Tecnai F20, Hillsboro, OR, USA). Nitrogen adsorption–desorption isotherms were recorded by Micromeritics ASAP 2460 adsorption apparatus at 77 K (ASAP 2460, Micromeritics, Norcross, GA, USA). The magnetic property of the sample was measured using VSM (LakeShore, Westerville, OH, USA). XPS was conducted at room temperature on an ESCALAB Xi+ instrument (Thermo Fisher Scientific, Waltham, MA, USA) equipped with an Al Kα source.

### 2.4. Adsorption of RhB

The adsorption experiments were carried out in a thermostated mechanical shaker and the speed was set at 200 rpm. A certain amount of the prepared adsorbents was added to 25 mL of RhB dye solution in a 50mL centrifuge tube. The effects of adsorbent dosage (0–50 mg), adsorption time (0–180 min), temperature (15–35 °C) and initial concentration (100–1500 mg/L) on adsorption were examined. After the adsorption experiment, about 3 mL of the mixture was sampled and filtered through a 0.45 mm membrane filter. The concentration of RhB was determined using Lambda 365 UV/Vis spectrophotometer (PerkinElmer, Waltham, MA, USA) at a wavelength of 554 nm. The removal efficiency R (%), the quantity of RhB adsorbed on the adsorbent Q_t_ (mg/g) and adsorption capacity Q_e_ (mg/g) were calculated using Equations (1)–(3).

(1) $$R\%=\frac{C_{0}-C_{t}}{C_{0}}\times 100\%$$

(2) $$Q_{t}\left(\mathrm{mg}/g\right)=\frac{C_{0}-C_{t}}{m}\times V$$

(3) $$Q_{e}\left(\mathrm{mg}/g\right)=\frac{C_{0}-C_{e}}{m}\times V$$

where C_0_ (mg/L) is the initial concentration, C_t_ (mg/L) is residual concentration at certain time and C_e_ (mg/L) is the equilibrium concentration; V (L) is the volume of dye solution and m (g) is the mass of adsorbent. To guarantee the reliability of data, all of the above experiments were performed in triplicate.

## 3. Results and Discussion

### 3.1. Characterizations of the Synthesized Biochars

**Figure 1:**
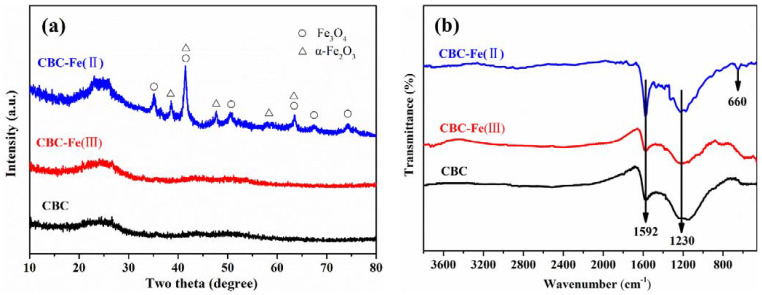
(**a**) XRD patterns and (**b**) FTIR spectrum of CBC, CBC-Fe(III) and CBC-Fe(II).

The XRD patterns of CBC, CBC-Fe(III) and CBC-Fe(II) are shown in Figure 1a. The broad diffraction peak at about 25° confirmed the presence of amorphous biochar in the three samples [39]. However, only the sample of CBC-Fe(II) showed the peaks related to iron oxide, which were identified as Fe_3_O_4_ and α-Fe_2_O_3_. Specifically, the diffraction peaks at 2θ = 35.1°, 41.4°, 50.4°, 62.9°, 67.2° and 74.1° corresponded to the (220), (311), (400), (422), (511) and (440) planes of Fe_3_O_4_, respectively. The diffraction peaks at 2θ = 38.7°, 41.4°, 47.8°, 58.2° and 63.7° corresponded to the (104), (110), (113), (024) and (116) planes of α-Fe_2_O_3_, respectively. The results implied that during the process of successive hydrothermal-pyrolyzing, the addition of Fe(II) salt promoted the formation of iron oxide particles, whereas the addition of Fe(III) salt had no positive effect on the formation of iron species in the biochar.

The functional groups of CBC, CBC-Fe(III) and CBC-Fe(II) were determined by FTIR spectra and are shown in Figure 1b. For the three samples, the peaks at 1592 cm^−1^ were attributed to the aromatic C=C groups stretching variations [27,40]. The peaks at 1230 cm^−1^ corresponded to the C–O stretching vibration [41,42]. Notably, the representative absorption bands for the stretching Fe–O band at around 660 cm^−1^ appeared for the sample of CBC-Fe(II) [43,44], further confirming that iron oxides had been successfully modified onto biochar.

**Figure 2:**
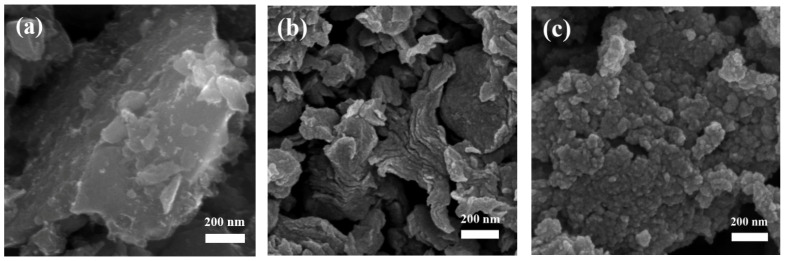
SEM images of (**a**) CBC, (**b**) CBC-Fe(III) and (**c**) CBC-Fe(II).

**Figure 3:**
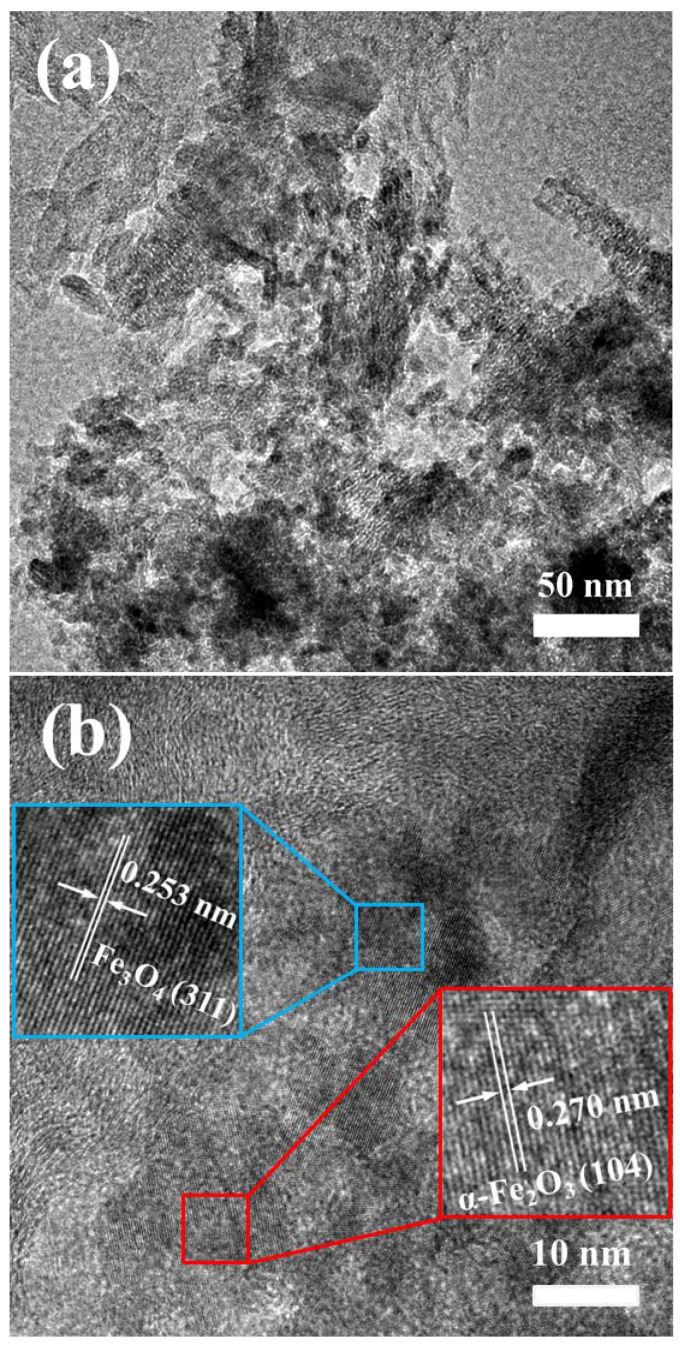
(**a**) TEM and (**b**) HRTEM images of CBC-Fe(II).

Figure 2 shows the typical morphologies of CBC, CBC-Fe(III) and CBC-Fe(II). It can be seen that the surface morphologies of biochars were obviously different. Compared with CBC-Fe(III) and CBC-Fe(II), CBC exhibited larger carbon particles. It was due to the fact that the modification of Fe could prevent the aggregation of carbon particles during biomass carbonization process. It can be seen that some nanostructured particles were observed for the sample of CBC-Fe(II), which could be attributed to the formation of nan iron oxides particles. TEM images of CBC-Fe(II) (Figure 3a,b) revealed that Fe_3_O_4_ and α-Fe_2_O_3_ nanoparticles were presented in biochar. Those nano iron oxides could give more active sites to the prepared biochar, which was in favor of dye adsorption. Figure 3b shows the exhibited characteristic fringes with lattice spacing of 0.253 nm and 0.270 nm, which could be indexed as the (311) plane of Fe_3_O_4_ and (104) plane of α-Fe_2_O_3_, respectively. The result was consistent with the results of XRD analysis as mentioned above.

**Figure 4:**
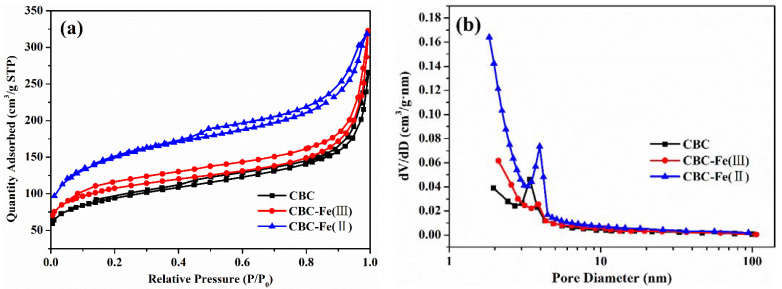
(**a**) N_2_ adsorption–desorption isotherms and (**b**) pore size distributions of CBC, CBC-Fe(III) and CBC-Fe(II).

N_2_ adsorption-desorption isotherms of CBC, CBC-Fe(III) and CBC-Fe(II) were detected and are shown in Figure 4a, and calculated pore parameters are summarized in Table 1. According to IUPAC classification, N_2_ adsorption–desorption isotherms of the three samples could be classified as type IV, which indicated that the mesoporous structure existed in the biochars. BET surface areas calculated were 338.6, 350.2 and 527.6 m²/g for CBC, CBC-Fe(III) and CBC-Fe(II), respectively. Notably, compared with CBC and CBC-Fe(III), the BET surface area of CBC-Fe(II) significantly increased. It was due to the fact that the pore structure was increased by the iron oxides formed on CBC-Fe(II). The high specific surface area for CBC-Fe(II) suggested the abundant accessibility of active sites on the adsorbent surface, which could be accountable for an increase in the adsorption efficiency for dye removal. As seen in Table 1, the average pore sizes of CBC, CBC-Fe(III) and CBC-Fe(II) were 4.855, 5.702 and 3.614 nm, respectively, which implied that the prepared samples were mesoporous materials with a certain amount of microporous structure. The result was consistent with the pore size distributions in Figure 4b. It was also observed in Table 1 that CBC-Fe(II) possessed the highest total pore volume and micropore volume compared with the other two samples, further confirming the porous property of CBC-Fe(II).

**Figure 5:**
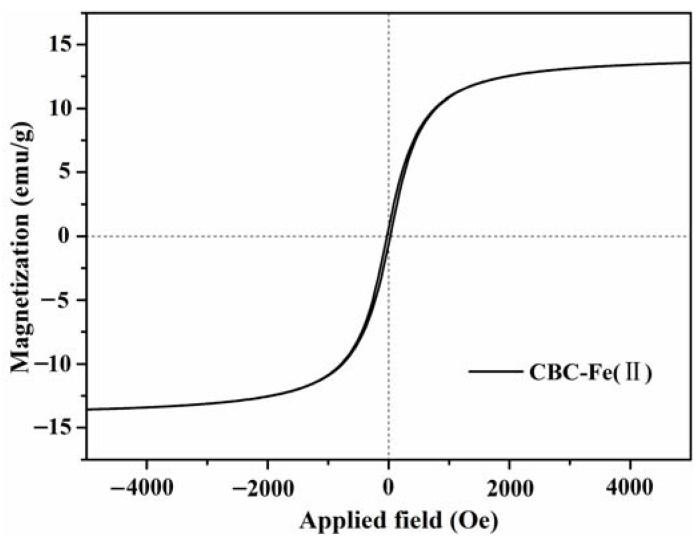
Magnetic hysteresis curves of CBC-Fe(II).

The magnetic behavior of the CBC-Fe(II) adsorbent was studied. The magnetic hysteresis curves (Figure 5) show that the saturation magnetization (Ms) of CBC-Fe(II) was determined to be 13.7 emu/g. It was concluded that the magnetization of CBC-Fe(II) was high enough to be magnetically collected simply by a magnet.

**Figure 6:**
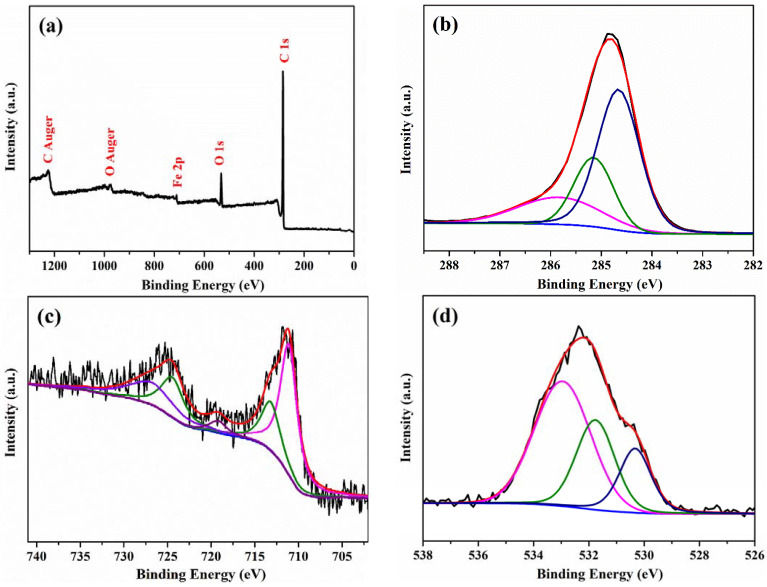
(**a**) XPS survey spectrum; high-resolution XPS spectra of (**b**) C 1s; (**c**) Fe 2p and (**d**) O 1s for CBC-Fe(II).

The XPS spectra of CBC-Fe(II) adsorbent were obtained and are shown in Figure 6. All of the peaks of the scan survey spectra in Figure 6a were ascribed to C, O and Fe elements. The deconvolutions of the C1s spectra of CBC-Fe(II) are shown in Figure 6b. The strongest peak located at 284.8 eV could be assigned to C–C. The other peaks at 285.2 and 285.9 eV corresponded to C–O and C=O functional groups, respectively [45]. Figure 6c represents the XPS spectra of Fe 2p. The peaks at 710.9 and 724.6 eV were respectively ascribed to 2p_3/2_ and 2p_1/2_ of Fe(II), whereas the peaks at 712.6 and 726.3 eV corresponded to 2p_3/2_ and 2p_1/2_ of Fe(III) [46]. The peak at 719.5 eV was attributed to the binding energy of the shake-up satellite [47]. The above results verified the existence of Fe_2_O_3_ and Fe_3_O_4_ in the sample of CBC-Fe(II), which was consistent with the XRD results. Figure 6d shows that the O1s peak was asymmetric and can be fitted into three peaks. The lowest peak, located at 530.3 eV, was ascribed to the lattice oxygen (O^2−^) of Fe_2_O_3_ or Fe_3_O_4_ in the CBC-Fe(II) sample [48]. The peak centered at 531.8 eV was attributed to C=O in the composite, while the highest peak, at 533.0 eV, was associated with C–O in the composite [45].

### 3.2. Batch Adsorption Studies

#### 3.2.1. Effect of Adsorbent Dosage on Adsorption of RhB

**Figure 7:**
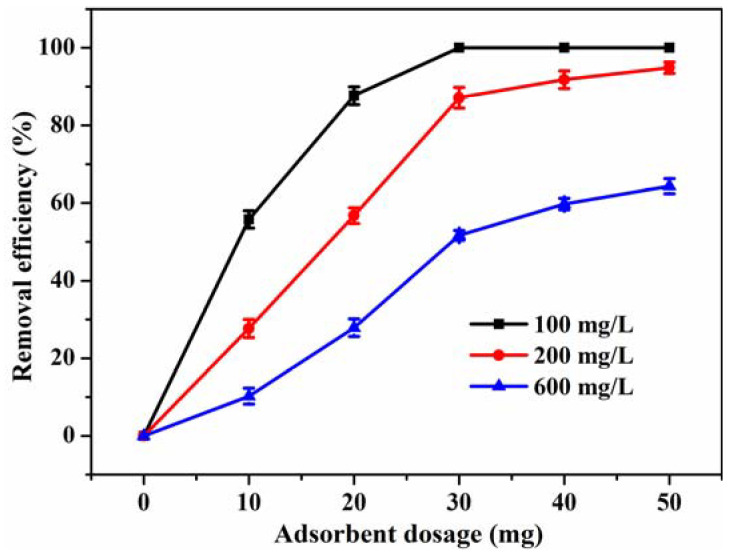
The influence of adsorbent dosage on removal efficiency of RhB by CBC-Fe(II).

To find the optimal adsorbent dosage, the effect of adsorbent dosage on removal efficiency of RhB by CBC-Fe(II) was investigated using 100, 200 and 600 mg/L as the initial dye concentrations. From the results presented in Figure 7, it can be seen that for various initial dye concentrations, the removal efficiency increased rapidly with increasing amounts of CBC-Fe(II) from 10 mg to 30 mg. It was because more accessible reactive sites are available to facilitate the dye adsorption process. However, when further increasing the amount of adsorbent from 30 mg to 50 mg, the required adsorption sites were getting closer to saturation, thus the removal efficiency only increased slightly. Thus, considering both the efficient removal of dye molecules and economic benefits, the optimal amount of the CBC-Fe(II) dosage was 30 mg.

#### 3.2.2. Effect of Contact Time on Adsorption of RhB

**Figure 8:**
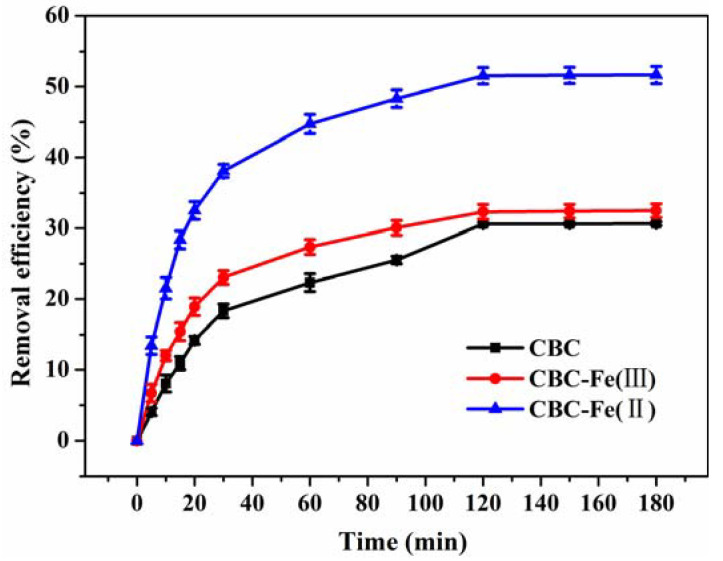
The influence of contact time on removal efficiency of RhB by CBC, CBC-Fe(III) and CBC-Fe(II) (adsorbent dosage: 30 mg; initial dye concentration: 600 mg/L).

Figure 8 shows the influence of reaction time on the removal efficiency of RhB by CBC, CBC-Fe(III) and CBC-Fe(II) under the same reaction temperature. The results obtained revealed that the removal efficiency of CBC-Fe(II) to RhB was much higher than that of CBC and CBC-Fe(III), demonstrating that CBC-Fe(II) possessed a relatively strong driving force on RhB. It was evidently observed that the removal efficiency of the three samples to RhB increased quickly during the initial stage, which was attributed to the free adsorption sites available for a large number of dye molecules. However, with the increase in contact time, the number of adsorption positions gradually decreased, leading to a slower adsorption rate. Finally, the adsorption equilibrium was achieved after 120 min, when the removal efficiency became almost constant.

#### 3.2.3. Effect of Temperature on Adsorption of RhB

**Figure 9:**
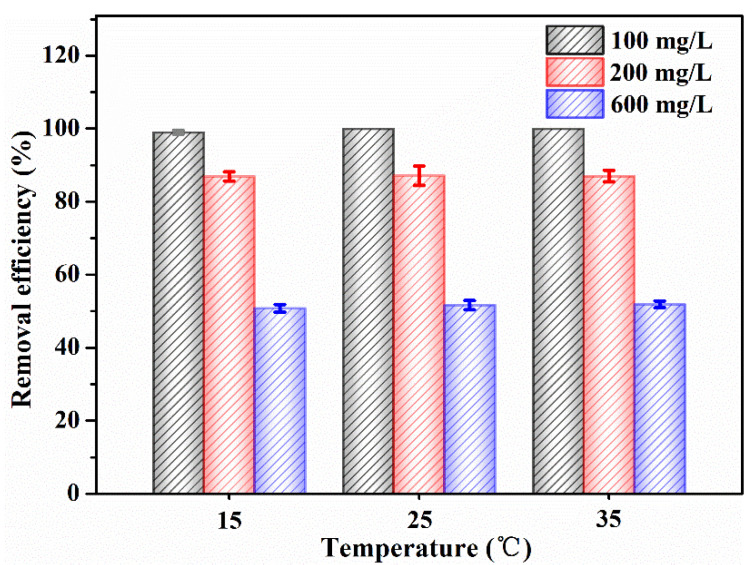
The influence of temperature on removal efficiency of RhB by CBC-Fe(II) (adsorbent dosage: 30 mg).

Figure 9 exhibited the effect of temperature on removal efficiency of RhB using CBC-Fe(II). It could be facilely observed that the removal efficiency of CBC-Fe(II) towards RhB remained the same at different temperatures (15 °C, 25 °C and 35 °C). Therefore, for the sake of saving energy and costs, 25 °C was chosen as the adsorption temperature throughout the whole experiment.

### 3.3. Kinetic and Isotherm Adsorption

#### 3.3.1. Adsorption Kinetic Models

**Figure 10:**
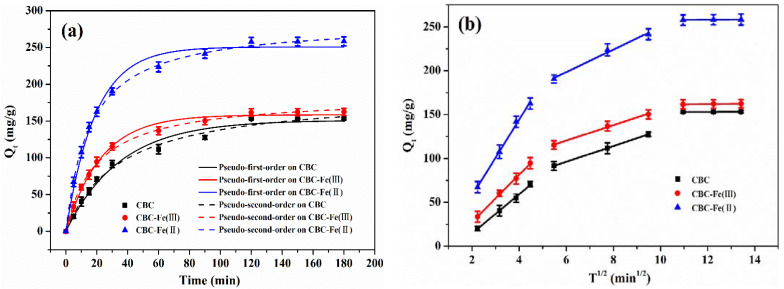
(**a**) Pseudo-first-order and pseudo-second-order and (**b**) intra-particle diffusion models of RhB adsorption by CBC, CBC-Fe(III) and CBC-Fe(II).

The adsorption kinetics of RhB onto CBC, CBC-Fe(III) and CBC-Fe(II) were investigated using pseudo-first-order and pseudo-second-order kinetic models, and the results are shown in Figure 10a. The adsorption kinetic models can be expressed as Equations (4) and (5), respectively:

(4) $$Q_{t}={\;Q}_{e}\left(1-\exp\left(-K_{1}t\right)\right)$$

(5) $$Q_{t}=\frac{K_{2}Q_{e}^{2}t}{1+K_{2}Q_{e}t}$$

where Q_e_ (mg/g) and Q_t_ (mg/g) represent the quantity of RhB adsorbed on the adsorbent at the equilibrium and arbitrarily given time t (min), respectively. K_1_ (1/min) and K_2_ (g/(mg∙min)) are adsorption rate constants of the pseudo-first-order and pseudo-second-order, respectively.

The related kinetic parameters of the above two models were obtained and are listed in Table 2. It was observed that for the three samples, the coefficients of correlation (R^2^) were higher for the pseudo-second-order than that of the pseudo-first-order kinetic model, indicating that the pseudo-second-order kinetic model was more appropriate to describe the adsorption behavior towards RhB. The result implied that the dye adsorption process by the biochars was mainly dominated by chemical adsorption. Moreover, the theoretical adsorption capacity of CBC-Fe(II) was 284.9 mg/g, which was much higher than that of CBC (186.3 mg/g) and CBC-Fe(III) (184.1 mg/g), showing the best adsorption performance of CBC-Fe(II).

Additionally, the intra-particle diffusion model (Figure 10b) was used to further evaluate the different stages of adsorption rate, which can be expressed as Equation (6).

(6) $$Q_{t}=K_{\mathrm{id}}t^{\frac{1}{2}}+c$$

where Q_t_ (mg/g) represents the quantity of RhB adsorbed on the adsorbent at arbitrarily given time t (min). K_id_ (mg/(g∙min^1/2^)) is the diffusion rate constant. Constant c is the thickness of boundary layer. 

From Figure 10b and Table 3, it is shown that for the three samples, all the lines did not pass through the origin, indicating that the intra-particle diffusion process was not the only rate-controlling mechanism for the adsorption system. The RhB adsorption process was comprised by three stages. In the first stage, the sharp rise was due to the liquid film diffusion process, while the second stage ascended more slowly, which represented the intra-particle diffusion process. The last equilibrium stage was attributed to the adsorption of dye molecules on the interior surface of adsorbents.

#### 3.3.2. Adsorption Isotherm Models

**Figure 11:**
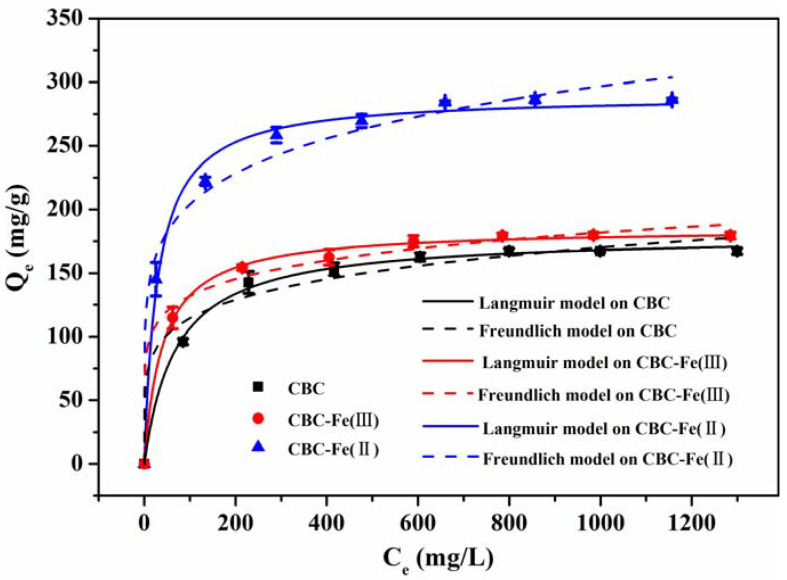
Langmuir and Freundlich isotherms of RhB adsorption by CBC, CBC-Fe(III) and CBC-Fe(II).

In order to study the interactive behavior between adsorbates and adsorbents, the equilibrium adsorption isotherms of RhB adsorption by CBC, CBC-Fe(III) and CBC-Fe(II) were described by the Langmuir and Freundlich isotherm models, and the results are presented in Figure 11. The two isotherm models can be expressed as Equations (7) and (8).

(7) $$Q_{e}=\frac{Q_{m}K_{l}C_{e}}{1+K_{L}C_{e}}$$

(8) $$Q_{e}=K_{F}C_{e}^{1/n}$$

where C_e_ (mg/L) is the equilibrium concentration of dye solution, Q_e_ (mg/g) and Q_m_ (mg/g) represent equilibrium adsorption capacity and maximum adsorption capacity of CBC-Fe(II), respectively. K_L_ (L/mg) and K_F_ [(mg/g)(mg/L)^1/n^] represent the constants of the Langmuir and Freundlich isotherms and n is the linearity index of adsorption intensity. 

It was observed from Figure 11 that the quantity of RhB adsorbed by CBC, CBC-Fe(III) and CBC-Fe(II) increased rapidly at low concentrations, then gradually approached the saturated adsorption capacity. The experimental data were fitted by the Langmuir and the Freundlich models, and the calculated parameters are described in Table 4. Apparently, for the three samples, the significant correlation coefficient value of Langmuir isotherm model was higher than that of the Freundlich isotherm model. The result indicated that the Langmuir isotherm model was superior for revealing the adsorption process, suggesting the equilibrium adsorption mechanism of RhB by CBC, CBC-Fe(III) and CBC-Fe(II) was more inclined to monolayer adsorption. Furthermore, the maximum adsorption capacity (Q_m_) for CBC, CBC-Fe(III) and CBC-Fe(II) obtained by the Langmuir model was 179.7 mg/g, 185.1 mg/g and 289.6 mg/g, respectively, which were close to the experimental values of 167.4 mg/g, 179.7 mg/g and 286.4 mg/g, respectively. It is worth noting that the CBC-Fe(II) adsorbent prepared in this study was found to show obvious advantages compared with biochars for RhB removal systhesized in other studies (Table 5).

### 3.4. Regeneration and Reusability of CBC-Fe(II)

**Figure 12:**
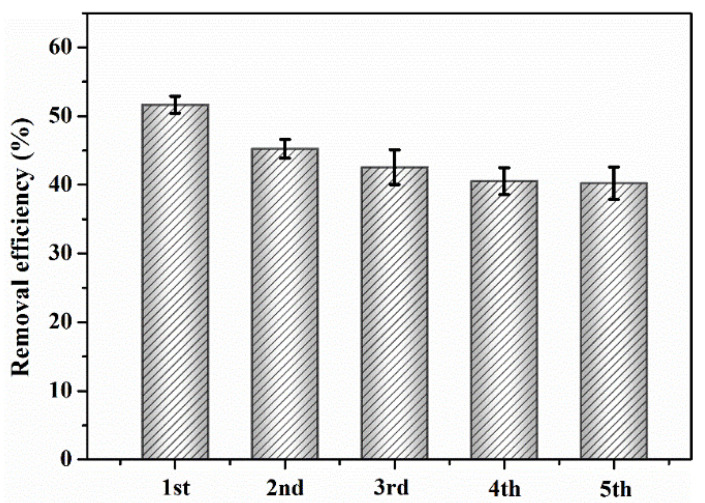
Recycling test of RhB adsorption by CBC-Fe(II) (adsorbent dosage: 30 mg, initial dye concentration: 600 mg/L).

To investigate the regeneration and reusability of CBC-Fe(II), the adsorption experiment was conducted in five consecutive cycles. After each adsorption experiment, the adsorbent was recollected by a magnet and washed with absolute ethyl alcohol and ultrapure water, respectively. For the next adsorption cycle, 25 mL of RhB solution was added to CBC-Fe(II). The results of repetitive use of the adsorbent for RhB removal are shown in Figure 12. It can be seen that the regeneration efficiency of the CBC-Fe(II) decreased with each recycling process, which might be due to the increased RhB residual on the adsorbent surface. However, CBC-Fe(II) still possessed a certain adsorption capacity towards RhB after five adsorption cycles.

## 4. Conclusions

In summary, a magnetically separable nano iron oxide–modified biochar (CBC-Fe(II)) was fabricated via a facile hydrothermal-pyrolyzing method. The synthesized CBC-Fe(II) adsorbent with a high surface area exhibited excellent magnetic properties and could be simply collected by a magnet. Compared with CBC and CBC-Fe(III), CBC-Fe(II) showed powerful adsorption performance for removing RhB from water. The RhB was optimally adsorbed at a CBC-Fe(II) dosage of 30 mg, with a contact time of 120 min and temperature of 25ºC. The adsorption process of RhB onto CBC-Fe(II) was found to obey the pseudo-second-order model and the Langmuir isotherm model well. Furthermore, the fitting results of the intra-particle diffusion model indicated that intraparticle diffusion was not the sole rate-controlling step as liquid film diffusion and interior surface adsorption also regulated the adsorption rate. All the results revealed that the prepared CBC-Fe(II) adsorbent possessed high potential in environmental remediation for dye removal.

## Figures and Tables

**Table 1:** BET surface area and pore parameters of CBC, CBC-Fe(III) and CBC-Fe(II).

Sample	S_BET_ (m^2^/g)	S_micro_ (m^2^/g)	V_total_ (cm^3^/g)	V_micro_ (cm^3^/g)	D_average_ (nm)
CBC	338.6	33.6	0.411	0.010	4.855
CBC-Fe(III)	350.2	99.9	0.477	0.057	5.702
CBC-Fe(II)	527.6	169.3	0.499	0.073	3.614

**Table 2:** Kinetic models parameters for RhB adsorption by CBC, CBC-Fe(III) and CBC-Fe(II).

Sample	Q_e_, Experimental (mg/g)	Pseudo-First-Order			Pseudo-Second-Order		
		K_1_ (1/min)	Q_e_, Model (mg/g)	R^2^	K_2_ (g/(mg∙min))	Q_e_, Model (mg/g)	R^2^
CBC	153.2	0.028	151.1	0.985	0.000154	186.3	0.992
CBC-Fe(III)	162.4	0.044	158.5	0.993	0.000273	184.1	0.998
CBC-Fe(II)	258.3	0.053	250.7	0.990	0.000226	284.9	0.999

**Table 3:** Intra-particle diffusion model parameters for RhB adsorption by CBC, CBC-Fe(III) and CBC-Fe(II).

Sample	First Linear Segment		Second Linear Segment		Third Linear Segment	
	K_id_ (mg/(g∙min^1/2^))	R^2^	K_id_ (mg/(g∙min^1/2^))	R^2^	K_id_ (mg/(g∙min^1/2^))	R^2^
CBC	22.339	0.995	8.965	0.999	0.074	0.905
CBC-Fe(III)	26.962	0.998	8.783	0.994	0.348	0.994
CBC-Fe(II)	43.337	0.996	12.831	0.981	0.224	0.998

**Table 4:** Parameters derived from isotherms of RhB adsorption by CBC, CBC-Fe(III) and CBC-Fe(II).

Sample	Langmuir			Freundlich		
	Q_m_ (mg/g)	K_L_ (L/mg)	R^2^	K_F_ [(mg/g)(mg/L)^1/n^]	n	R^2^
CBC	179.7	0.0145	0.998	51.785	5.808	0.972
CBC-Fe(III)	185.1	0.0250	0.998	69.732	7.212	0.988
CBC-Fe(II)	289.6	0.0343	0.992	96.384	6.144	0.982

**Table 5:** Comparison of the adsorption capacity of RhB with other biochar adsorbents reported in the literature.

Biochar	Biomass	Qm (mg/g)	References
biochar	cassava slag	105.3	[25]
BHC-800	bamboo shoot shell	85.8	[26]
sulfur-doped biochar	tapioca peel waste	33.1	[27]
earthworm manure derived biochar	earthworm manure	21.6	[49]
plantain peel activated biochar	plantain peel	84.4	[50]
magnetic chicken bone-based biochar	chicken bone	96.5	[51]
Fe–N co-modified biochar	coconut shell	12.4	[52]
magnetic biochar (AMBC)	Rice straw	53.7	[53]
CBC-Fe(II)	*Chlorella vulgaris*	286.4	This study

## Data Availability

The data presented in this study are available on request from the corresponding authors.
